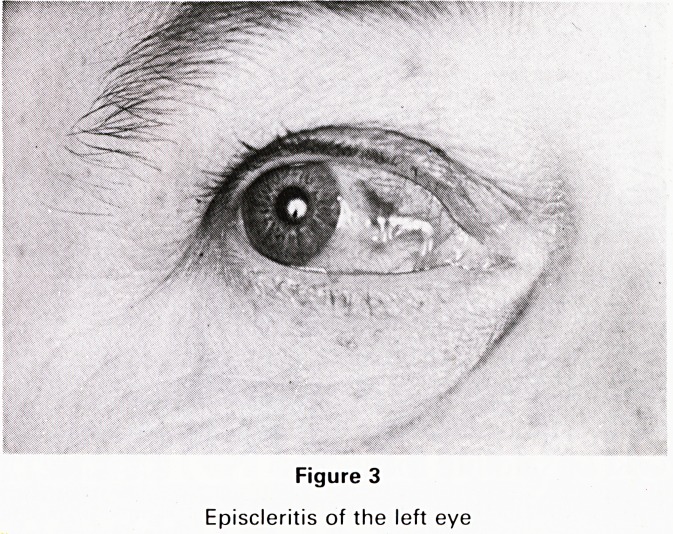# Sweet's Syndrome and Erythema Nodosum

**Published:** 1988-08

**Authors:** C. E. H. Grattan, C. T. C. Kennedy, S. C. Glover, R. J. Mann

**Affiliations:** Senior Registrar in Dermatology, The General Hospital, Birmingham; Consultant Dermatologist, Bristol Royal Infirmary; Consultant Physician and Specialist in Communicable Diseases, Ham Green Hospital, Bristol; Consultant Dermatologist, Princess Margaret Hospital, Swindon


### Sweet's syndrome and Erythema nodosum

**C. E. H. Grattan MB, MRCP**
Senior Registrar in Dermatology, The General Hospital, Birmingham
**C. T. C. Kennedy, MB, FRCP**
Consultant Dermatologist, Bristol Royal Infirmary
**S. C. Glover MB, FRCP (Ed)**
Consultant Physician and Specialist in Communicable Diseases, Ham Green Hospital, Bristol
**R. J. Mann MB, MRCP**
Consultant Dermatologist, Princess Margaret Hospital, Swindon

### INTRODUCTION

Sweet's syndrome (acute febrile neutrophilic dermatosis) is a fairly uncommon skin disorder characterised by acute painful dusky red plaques, sometimes with overlying papules, vesicles or pustules, usually situated on the head, neck and upper trunk. Associated fever and neutrophilia commonly occur. While this disorder is well known to Dermatologists, specialists in other fields may be less familiar with it. However, most physicians are familiar with erythema nodosum, which typically presents with tender nodules over the shins, resolving over weeks or months with a characteristic bruised appearance. Both conditions are thought to be reactive and both respond to corticosteroids. We have seen three cases where Sweet's syndrome and erythema nodosum have occurred together suggesting that there is a degree of overlap between these two disorders. Although this association has been recognised before [1,2] we are reporting these cases because we feel that many clinicians, who are not Dermatologists, may be unfamiliar with the clinical features of Sweet's syndrome.

### CASE 1

A 48-year-old woman became unwell with a severe sore throat which cleared with penicillin. Two weeks later, she became feverish and developed typical erythema nodosum over both shins (Fig. 1), tender red plaques on her neck, chest (Fig. 2) and arms and episcleritis of her left eye (Fig. 3). Her plasma viscosity was raised at 1.95cp,

**Figure 1**

Erythema nodosum on the shins

**Figure 2**

Painful dusky papules on the chest.

**Figure 3**

Episcleritis of the left eye but a white cell count and differential were normal. A throat swab, antistreptolysin O titre (ASOT), antinuclear factor (ANF), Rose-Waaler (RW) and 1:1000 Mantoux test were negative. Chest X-ray was normal. Skin biopsy of a chest lesion showed an intense neutrophilic infiltrate in the dermis, typical of Sweet's syndrome. The skin lesions on the upper half of her body resolved rapidly with prednisolone but she developed arthralgia in her wrists, right ankle and knee joints as the dose of steroid was reduced over the following week. Ten days later the erythema nodosum had also faded to a bruised appearance and her joints and eye were improving. After a further three weeks the steroids were withdrawn completely with no recurrence of the skin lesions, arthralgia or episcleritis.

### CASE 2

A 29-year-old man was treated with penicillin for a culture proven streptococcus pyogenes throat infection. Ten days after the onset he became pyrexial with myalgia and arthropathy of the large joints. Multiple red plaques, some surmounted by vesicles, appeared on his face, upper trunk, forearms and the backs of his hands together with typical erythema nodosum over his shins. His white cell count was elevated at $16.8 \times 10^9/1$ with 93% neutrophils, and the erythrocyte sedimentation rate (ESR) was 116 mm/hr. A chest X-ray was normal. Cultures of blood and urine, ANF, RF and tests for circulating immune complexes were negative. Histology of a skin biopsy from his trunk showed an intense neutrophilic infiltrate in the dermis. The fever and arthropathy responded to treatment with aspirin and indomethacin and the skin lesions resolved over two weeks without steroids.

### CASE 3

A 33-year-old man was admitted with acute otitis externa which was treated with amoxycillin and analgesics. A week later the antibiotic was changed to cephalexin following a recurrence of the earache and the onset of pyrexia and malaise. The next day he developed tender nodules on the shins and thighs, a vesicular plaque on his right forearm and some pustular lesions on his scalp. Ulcers were also noted over the posterior pharynx. Swabs from the skin lesions, throat and ear were negative. The white cell count was raised at $15.5 \times 10^9/1$ with 70% neutrophils and the ESR was 60 mm/hr. A skin biopsy from his right forearm showed a marked dermal infiltrate of polymorphs with leucocyte debris and lymphocytes. Following a further course of amoxycillin with flucloxacillin the pyrexia settled and the skin and oral lesions healed.

### DISCUSSION

These three cases all show the common features of fever, malaise and skin lesions following infection. Case 1 also had episcleritis which may occur with Sweet's syndrome. It should be noted that Cases 2 and 3 resolved without steriods, emphasising the usual self-limiting and reactive course of the condition.

A diagnosis of Sweet's syndrome should be suspected when painful skin lesions erupt acutely on a background of fever and malaise, even if 'typical' erythema nodosum is present on the lower extremities. It should usually be possible to distinguish the two conditions on skin biopsy because the infiltrate in Sweet's is predominantly dermal and neutrophilic but in erythema nodosum it is characteristically localised to the subcutaneous tissue, and lymphocytes and histiocytes predominate in the chronic forms.

As well as occurring as a reactive disorder to infection it should be noted that Sweet's syndrome has also been described with acute leukaemia [3], and there have been several reports linking it to visceral and metastatic carcinoma so the possibility of underyling malignant disease should not be forgotten.

### REFERENCES

1. SPATZ, S. A. (1985) Erythema nodosum in Sweet's syndrome. *Cutis* **35**, 327–330.
2. BLAUSTEIN, A., MORENO, A., NOGUERA, J., and DE MORAGAS, J. M. (1985) Septal granulomatous panniculitis in Sweet's syndrome – report of two cases. *Archives of Dermatology* **121**, 785–788.
3. MATTA, M., MALAK, J., TABET, E. and KURBAN, A. K. (1973) Sweet's syndrome: systemic associations. *Cutis* **12**, 561–565.

## Figures and Tables

**Figure 1 f1:**
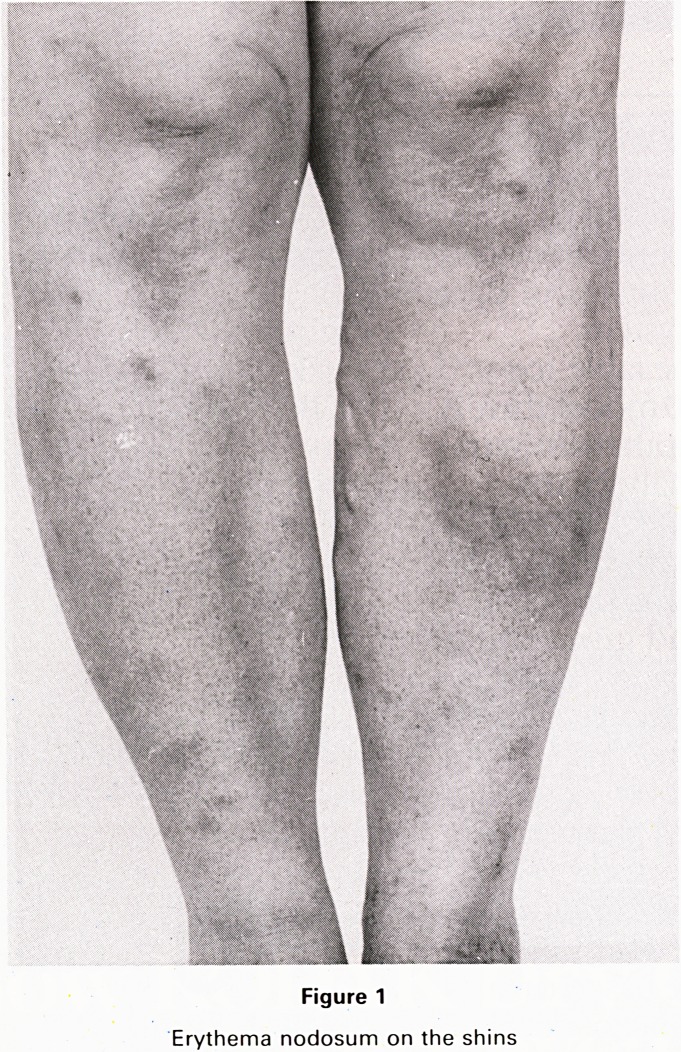


**Figure 2 f2:**
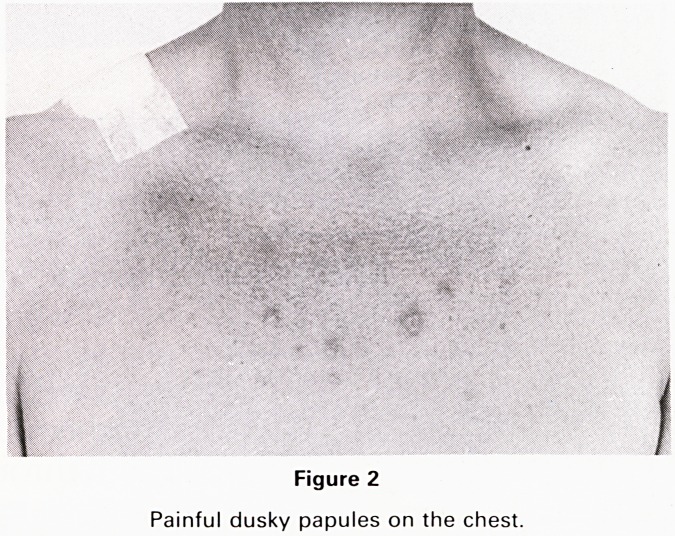


**Figure 3 f3:**